# Isoprene Responses and Functions in Plants Challenged by Environmental Pressures Associated to Climate Change

**DOI:** 10.3389/fpls.2017.01281

**Published:** 2017-07-26

**Authors:** Alessio Fini, Cecilia Brunetti, Francesco Loreto, Mauro Centritto, Francesco Ferrini, Massimiliano Tattini

**Affiliations:** ^1^Department of Agricultural and Environmental Sciences – Production, Landscape, Agroenergy, University of Milan Milan, Italy; ^2^Department of Biology, Agriculture and Food Science, National Research Council of Italy, Trees and Timber Institute Sesto Fiorentino, Italy; ^3^Department of Agrifood Production and Environmental Sciences, University of Florence Florence, Italy; ^4^Department of Biology, Agriculture and Food Science, National Research Council of Italy Rome, Italy; ^5^Department of Biology, Agriculture and Food Science, National Research Council of Italy, Institute for Sustainable Plant Protection Sesto Fiorentino, Italy

**Keywords:** climate change, drought and heat stress, fast-growing plants, isoprene biosynthesis vs. isoprene emission, membrane protection, stomatal conductance

## Abstract

The functional reasons for isoprene emission are still a matter of hot debate. It was hypothesized that isoprene biosynthesis evolved as an ancestral mechanism in plants adapted to high water availability, to cope with transient and recurrent oxidative stresses during their water-to-land transition. There is a tight association between isoprene emission and species hygrophily, suggesting that isoprene emission may be a favorable trait to cope with occasional exposure to stresses in mesic environments. The suite of morpho-anatomical traits does not allow a conservative water use in hygrophilic mesophytes challenged by the environmental pressures imposed or exacerbated by drought and heat stress. There is evidence that in stressed plants the biosynthesis of isoprene is uncoupled from photosynthesis. Because the biosynthesis of isoprene is costly, the great investment of carbon and energy into isoprene must have relevant functional reasons. Isoprene is effective in preserving the integrity of thylakoid membranes, not only through direct interaction with their lipid acyl chains, but also by up-regulating proteins associated with photosynthetic complexes and enhancing the biosynthesis of relevant membrane components, such as mono- and di-galactosyl-diacyl glycerols and unsaturated fatty acids. Isoprene may additionally protect photosynthetic membranes by scavenging reactive oxygen species. Here we explore the mode of actions and the potential significance of isoprene in the response of hygrophilic plants when challenged by severe stress conditions associated to rapid climate change in temperate climates, with special emphasis to the concomitant effect of drought and heat. We suggest that isoprene emission may be not a good estimate for its biosynthesis and concentration in severely droughted leaves, being the internal concentration of isoprene the important trait for stress protection.

## Why Isoprene Emission May Become More Relevant In a Drier and Warmer Climate?

Isoprene (2-methyl-1,3-butadiene), the major volatile organic compound (VOC) emitted by biogenic sources, has driven attention because of its impact on atmospheric chemistry and climate (Atkinson, 2000). Globally, 0.5–0.6 Pg C are emitted as isoprene annually, accounting for 50% of total biogenic volatile organic compounds (BVOCs), and for 30% of non-methane hydrocarbons emissions (Guenther et al., 2006, 2012). Isoprene is highly volatile and reactive, and its emission by terrestrial plants can substantially affect the concentration of tropospheric ozone (O_3_), the lifespan of methane, and the nucleation, condensation or coagulation of secondary aerosol(s) (Pike and Young, 2009; Ying et al., 2015). In the present global change scenario, isoprene emission (Iso_e_) is of major concern for several reasons.

First, urban population is expected to increase by approximately 70% by 2050 (United Nations, 2015), and growing megacities are hotspots of atmospheric gaseous and particulate pollutants, with economic, sanitary and social consequences (Baudic et al., 2016). For example, air pollution, particularly tropospheric O_3_ and particulate matter, was responsible of 34,143, and 17,800 excess deaths, in Italy and France, respectively, during 2010 (Global Burden of Disease [GBD], 2013; Mori et al., 2015; Baudic et al., 2016). While at null nitrogen oxide (NO_x_) concentration, isoprene can even lower tropospheric [O_3_], when levels of NO_x_ are high, a single isoprene molecule leads to the formation of several O_3_ molecules (Zeng et al., 2008). In urban areas, where NO_x_ concentration is high (the so-called NO_x_-saturation regime), O_3_ production is highly responsive to VOCs (Sillman, 1999; Deguillaume et al., 2008; Ling et al., 2014). Thus, to limit O_3_ pollution in NO_x_-saturated urban sites, policy actions aimed at reducing VOC emission may be more effective and easier to actuate than the policies aimed at decreasing NO_x_ concentration (Baudic et al., 2016; Khedive et al., 2017).

Second, conversion of isoprene-emitting forest to low-emitting cropland to match the increasing demand for food, globally decreased isoprene concentration by 15% during the last century (Lathiere et al., 2010). However, the ongoing shift to bioenergy crops (e.g., giant reed) and short rotation forests (e.g., poplar) will likely increase isoprene load, particularly at regional scale (Hardacre et al., 2013; Sharkey and Monson, 2014). For example, in South East Asia, the 27 Mha expansion of land cultivated with oil palm, which can emit three times more isoprene than the native crops (Fowler et al., 2011), increased surface O_3_ by 11% (Ashworth et al., 2012). Similarly, the expansion of short rotation forests (mainly poplar) in the temperate northern hemisphere triggers the increase in isoprene burden predicted for boreal Eurasia, North America, and China, where O_2_/O_3_ mixing ratios are expected to increase up to 2.26 ppb (Ashworth et al., 2012; Hardacre et al., 2013; Zenone et al., 2016).

Third, species from all taxonomic groups have spread around the world, mostly because of human activities. These biological invasions may alter the emission profile of volatiles. For instance, Llusià et al. (2010) have found a lower emission of isoprenoids in native species growing in Hawaii, compared to co-occurring alien species. This was attributed to the lower emission potential of native species relative to aliens, within any given phylogenetic line, though further research is required to upscale this phenomenon. Similarly, tree genera characterized by extensive speciation and hybridization have been reported to emit isoprene more frequently than their phylogenetically nearest non-speciose genera (Dani et al., 2014). Isoprene, being highly volatile (Henry’s law constant of 7,780 Pa m^3^ mol^-1^, Harley, 2013), is a ‘quick’ metabolite capable of improving photosynthetic performance under physiological (non-stressful) (Pollastri et al., 2014) and under transient, usually mild-to-moderate, stress conditions (Loreto and Fineschi, 2015; Maja et al., 2016). Furthermore, it provides protection against generalist pests (Llusià et al., 2010; Harrison et al., 2013); thus alien species, which lack specialist parasites, may greatly benefit from being emitters (Laothawornkitkul et al., 2008; Mithofer and Boland, 2012).

Finally, Iso_e_ is exponentially linked to temperature, thus global warming is expected to increase the load of volatile compounds (Fares et al., 2011; Lahr et al., 2015). Nonetheless, a conclusive picture of the effect of climate change on Iso_e_ and hence on the chemistry of the atmosphere is far from being drawn, as the wide range of co-occurring environmental factors (e.g., rising CO_2_) may have synergic or antagonistic effects on isoprene biosynthesis (Dieleman et al., 2012).

We focus our discussion on the effects of concomitant stress factors on the biosynthesis and emission of isoprene, with the aim of further exploring isoprene functional roles in hygrophylic plants challenged by ‘novel’ environmental pressures associated to climate change in temperate climates (e.g., Cfa, Cfb in Koppen Geiger classification).

## Exploring the Significance of Isoprene In Plants Challenged By Stress

The functional reasons for Iso_e_ are still a matter of debate (Sharkey and Monson, 2017). It was hypothesized that isoprene biosynthesis (Iso_s_) evolved as an ancestral mechanism in plants adapted to high water availability, to cope with transient and recurrent oxidative stresses during their water-to-land transition (Vickers et al., 2009; Loreto et al., 2014). Consistently the tight association between Iso_e_ and species hygrophily suggests that Iso_e_ may be a favorable trait to cope with occasional exposure to stresses in mesic environments (Harrison et al., 2013; Monson et al., 2013; Loreto et al., 2014). Instead, xeric evergreen species inhabiting harsher environments, which require constitutive emissions over a longer time-scale level, generally produce compounds less volatile than isoprene, such as monoterpenes and sesquiterpenes (Loreto and Fineschi, 2015). Fast-growing hygrophilous *Quercus* species, such as most North American and some European oaks (e.g., *Q. robur*) emit isoprene, whereas isoprene is replaced by monoterpenes in xeric oaks, such as *Q. ilex* and *Q. suber* (Loreto et al., 1998, 2009; Sharkey et al., 2008). This conforms the notion that marked differences in gene sequences encoding isoprene synthase have been found not only between plant groups, but also within each individual group (Dani et al., 2014), and suggests that environmental conditions may have contributed shaping the evolution of isoprenoid synthesis (Monson et al., 2013).

Several fast-growing, isoprene-emitting plants have moved to areas with harsher climate conditions than those of habitats they evolved (Owen et al., 2013). In many instances, extended periods of rainfall scarcity, which usually occur in combination with high both solar irradiance and air temperature may pose serious challenges to plant survival, not only to the profitable production of biomass. Furthermore, the suite of morpho-anatomical traits (e.g., low tissue density, thin cuticle, large vessels, high vein density, see Reich, 2014) does not allow a conservative water use in hygrophilic mesophytes and their ability to withstand combined stress conditions may greatly depend on the so-called metabolic plasticity, which mostly involves secondary metabolites (Tattini et al., 2015). There is evidence that the biosynthesis of isoprenoids is stimulated via ROS-signaling (Fanciullino et al., 2014). This may help explain why the biosynthesis of secondary metabolites, particularly of isoprene is generally uncoupled from photosynthesis (*A*_N_) in drought-stressed leaves (Affek and Yakir, 2003; Loreto and Schnitzler, 2010; Centritto et al., 2011).

The lack of correlation between *A*_N_ and isoprene biosynthesis/emission becomes clearer when plants concurrently face multiple stresses. Indeed, there is compelling evidence that carbon sources alternative to recently fixed CO_2_ may have particular significance when photosynthesis is constrained by stress (Brilli et al., 2007). These alternative carbon sources may include: non-structural carbohydrates (Kreuzwieser et al., 2002; Funk et al., 2004; Schnitzler et al., 2004); phosphoenolpyruvate imported from the cytosol (Rosenstiel et al., 2003; Fortunati et al., 2008; Jardine et al., 2010); re-fixation of respired CO_2_ (Loreto et al., 2004); isoprenoid precursors from the cytosolic mevalonate pathway (Flügge and Gao, 2005); photorespiratory carbon (Jones and Rasmussen, 1975). Carbon derived from photorespiration may have particularly value in sustaining Iso_s_ when plants experience intense drought and heat stresses (Jardine et al., 2014). Drought stress depresses photosynthesis to a greater extent than photorespiration (Atkin and Macherel, 2009), particularly at high temperatures (Centritto et al., 2011), while elevated temperatures enhance both the substrate (DMADP) availability and the activity of isoprene synthase (Rasulov et al., 2010). Air temperature mostly regulates Iso_s_ in plants growing at light intensities that saturate photosynthesis (Monson, 2002; Mayrhofer et al., 2005; Fares et al., 2011; Niinemets and Sun, 2015), since Iso_e_ does not saturate even at very high photosynthetic photon flux density (>2000 μmol m^-2^ s^-1^, Geron et al., 2006; Loreto and Schnitzler, 2010).

In plants concurrently experiencing water and heat stress, stomatal closure reduces latent heat and exacerbates sensible heat load (Tattini et al., 2015). In particular, hygrophilic isoprene-emitters steeply close stomata, even at moderate drought, to avoid tissue dehydration (Brilli et al., 2007; Tattini et al., 2015; Velikova et al., 2016). These are the conditions under which isoprene biosynthesis is largely stimulated. Isoprene has been reported to enhance drought resistance of many fast-growing species, including tobacco and poplars. In all cases, isoprene-emitting lines showed reduced depression of photosynthesis, and less oxidative damage than non-emitting lines, when exposed to drought (Ryan et al., 2014; Tattini et al., 2014; Vanzo et al., 2017).

## Isoprene Mode of Action: Facts and Speculations of An Open Debate

Because isoprene is costly for leaves (20 ATP and 14 NADPH for each molecule of isoprene produced by CO_2_ fixation through photosynthesis) (Sharkey and Yeh, 2001) the great investment of leaves for Iso_s_ under stressful conditions must have functional reasons (Sharkey and Monson, 2017). Isoprene may play multiple functions in countering the detrimental effects of supernumerary photons reaching the chloroplast, when the leaf ability to process radiant energy to carbon fixation is severely constrained by environmental stressors (Loreto and Schnitzler, 2010). Isoprene is effective in preserving the integrity of thylakoid membranes (Velikova et al., 2011, 2015). *Populus × canescens* lines where Iso_s_ is suppressed displayed reduced photosynthetic electron transport rate (ETR) during heat stress, and did not recover photosynthesis at the level of the corresponding isoprene-emitting lines after relief from stress (Behnke et al., 2007). The protective functions of isoprene on membrane-associated processes (also observed under ‘physiological’ conditions, Pollastri et al., 2014) may not depend simply on the hydrophobic interaction between isoprene and the lipid acyl chains of membranes (Siwko et al., 2007), as isoprene concentration inside membranes is too low to effectively modulate their bulk lipid phase (Harvey et al., 2015). Benefits for membrane stability associated to Iso_e_ may also result from both the up-regulation of proteins associated with photosynthetic complexes (Velikova et al., 2014) and the enhanced biosynthesis of relevant membrane components, such as mono- and di-galactosyl-diacyl glycerols and unsaturated fatty acids (Velikova et al., 2015). In simpler terms, isoprene-induced improvement in the use of radiant energy to carbon fixation may reduce the risk of photo-oxidative stress in isoprene-emitting leaves. Protection of photosynthetic membranes may be induced by isoprene indirectly, as isoprene is also known to scavenge reactive oxygen species (ROS) (Loreto and Velikova, 2001; Affek and Yakir, 2002; Velikova et al., 2004). The antioxidant effect of isoprene is especially clear in the case of singlet oxygen (^1^O_2_), the most dangerous ROS in chloroplasts. This effect was empirically demonstrated by Velikova et al. (2004), and has now been theoretically framed (Zeinali et al., 2016). ROS scavenging inside leaves explains the formation of isoprene oxidation products, mostly methyl-vinyl-ketone and methacrolein, in plants exposed to a wide range of stressors, especially heat, in both controlled (Jardine et al., 2012, 2013) and field conditions (Cappellin et al., 2017).

Despite the large body of evidence summarized above, there are open questions that still challenge the idea that isoprene might have a definite role in plant protection. Why did only about 20% of the plants worldwide develop the capacity to emit isoprene? (Loreto and Fineschi, 2015). Why are these plants spread all over biomes and climatic areas (Loreto and Fineschi, 2015), and are not concentrated where stress protection becomes more relevant for securing plant survival, growth, and reproduction?

In many instances, Iso_e_ increases under mild to moderate drought, but declines steeply when plants face severe drought (Brilli et al., 2007, 2013; Centritto et al., 2011; Tattini et al., 2014, 2015). Therefore, it has been hypothesized that isoprene plays a beneficial role only in response to mild stress, whereas non-volatile, more stable metabolites, produced through the same metabolic pathway of isoprene (the MEP pathway, i.e., carotenoids and abscisic acid), serve functions of greater significance when plants are challenged by severe stress. This is a revisited formulation of the “opportunistic hypothesis”, firstly postulated by Owen and Peñuelas (2005). For example, in *Xerophyta humilis*, Iso_e_ ceased at 5% RWC, but zeaxanthin replaced isoprene to enhance membrane stability, thus allowing prompt chloroplast re-assembly upon re-watering of this resurrection plant (Beckett et al., 2012). Recent evidence suggests that isoprene may serve antioxidant functions (*sensu lato*) of increasing significance in plants concurrently challenged by drought and heat. Indeed, the activities of primary antioxidants, such as antioxidant enzymes, and the concentration of zeaxanthin may decrease in high light-exposed plants during the hottest hours of the day, whereas biosynthesis and emission of isoprene are promoted in the same conditions (Brunetti et al., 2015; Tattini et al., 2015).

Isoprene emission might even have a regulatory role, differentially setting the flow of carbon in the MEP pathway along stress progression. The transient increase of isoprene biosynthesis/emission in drought-stressed leaves might serve to use of excess reducing power, limiting the accumulation of dimethylallyl diphosphate (DMADP) and its consequent feedback down-regulation of the whole MEP pathway (Banerjee et al., 2013; Ghirardo et al., 2014). Sustained isoprene formation under stress conditions may also indirectly contribute to increase the carbon flux into the MEP pathway leading to the *de novo* biosynthesis of foliar abscisic acid, the stress hormone controlling stomatal aperture in drying soil to prevent water loss (Zhang and Davies, 1987). This effect might be exacerbated when plants concurrently face high solar irradiance and temperatures, which are known to increase the availability of DMADP (Mayrhofer et al., 2005; Sharkey and Monson, 2014). Thus, internal isoprene concentration (not isoprene emission) might ‘prime for drought stress response,’ triggering a general protective function that also involves changes in non-volatile isoprenoids, soluble carbohydrates and phenylpropanoids (Tattini et al., 2014). This may sustain the need of large metabolic adjustments of hygrophylic plants suddenly facing the unpredictable pressures imposed by “anthropogenic” planting sites, where microclimates can be very different from those where these species have evolved.

## Is Isoprene Emission a Good Proxy of Internal Isoprene and of Plant Stress Response?

Isoprene emission has been usually taken as a good estimate of Iso_s_, but Iso_e_ might largely differ from Iso_s_, e.g., as consequence of drought-induced declines in stomatal conductance (*g*_s_). It has been hypothesized that stomata cannot control the emission of VOCs with high Henry’s low constant, such as isoprene, even during rapid reductions in *g_s_* (Niinemets and Reichstein, 2003). If Iso_s_ remains constant or even increases when stress induces stomatal closure, then the increased gradient between the internal and external concentration of isoprene should compensate for the increased resistance to isoprene outflow. However, under chronic or severe reductions of *g*_s_, isoprene concentration inside the leaf (Iso_i_) largely exceeds Iso_e_ (Velikova et al., 2016), and Iso_i_ might represent a more suitable estimate of Iso_s_ compared to Iso_e_ (Brunetti et al., 2015; Tattini et al., 2015).

It has been also speculated that lipid membranes are saturated with isoprene even at low emission rates (because isoprene is highly hydrophobic in its nature), and that any increase in Iso_s_ will increase isoprene diffusion through membranes rather than enhancing its membrane concentration (Vickers et al., 2009). However, recent results discussed above revisited this concept and showed that isoprene concentration in membranes is generally low (Harvey et al., 2015). Therefore, it cannot be excluded that steep reductions of *g*_s_ may induce large accumulation of isoprene inside leaves, on a short time-scale, thereby altering membrane composition (Velikova et al., 2015), while providing efficient antioxidant and priming functions.

**FIGURE 1:**
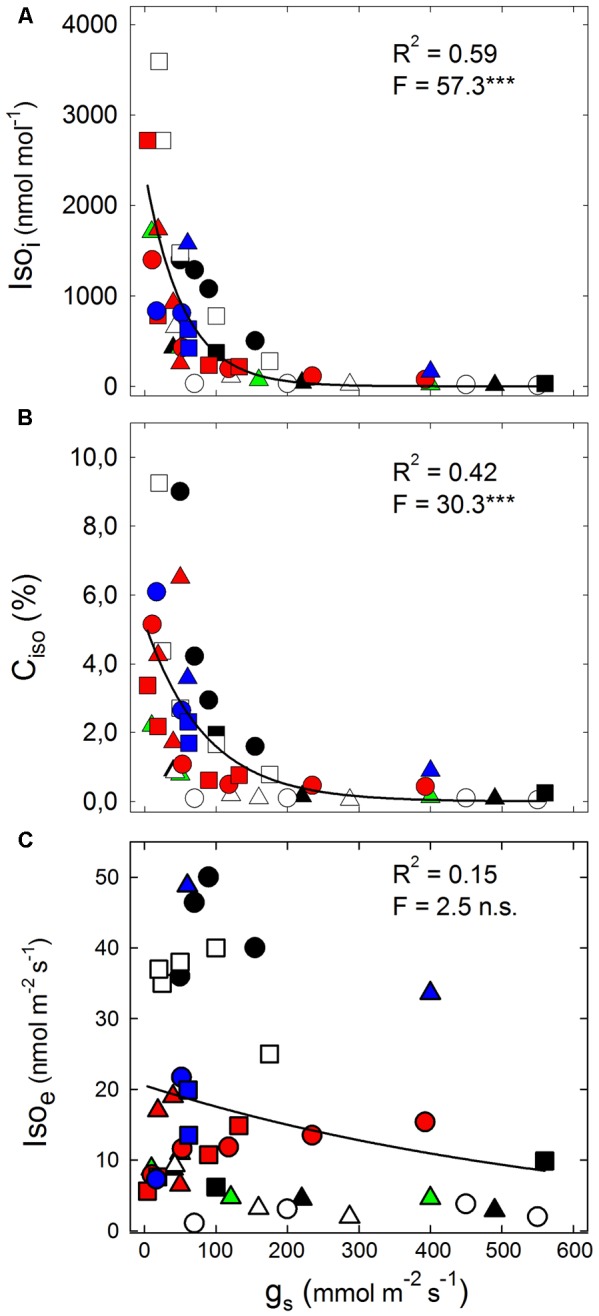
The relationship between **(A)** internal isoprene concentration (Iso_i_); **(B)** percent of fresh assimilated carbon lost as isoprene emission (C_iso_); **(C)** isoprene emission (Iso_e_) and stomatal conductance (*g*_s_). Isoprene concentration was calculated using a simplified version of the equation proposed by Singsaas et al. (1997), as Iso_i_ = 2.83 × Iso_e_/*g*_s_, where the factor 2.83 is the ratio of the diffusion coefficient of water vapor through air to that of isoprene through air; C_iso_ = 5 × (Iso_e_, μmol m^-2^ s^-1^)/(*A*_N_, μmol m^-2^ s^-1^) × 100. Non-linear correlations have been drawn using the following exponential decay curve, Iso_x_ = a ^-b ×χ^. Data points derive from the experimental data reported in: Brilli et al. (2007) (*Populus alba:* 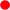); Brilli et al. (2013) (*Eucalyptus citriodora*: 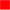); Funk et al. (2004) (*P. deltoides:* 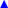); Tani et al. (2011) (*Quercus serrata:* 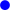); Tattini et al. (2014) (*Nicotiana tabacum:* ○); Tattini et al. (2015) (*Platanus × acerifolia:* ●); Velikova et al. (2016) (*Arundo donax:* ▄); Pegoraro et al. (2004) (*Q. virginiana:* 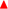); Dani et al. (2014) (▴, *E. occidentalis* and *E. camaldulensis:* 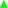); Staudt et al. (2016) (*Q. pubescens:* 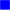); Marino et al. (2017) (*P. nigra:* □); Brunetti et al., personal communication, (*Moringa oleifera:* Δ).

The decline in *g*_s_ is a good proxy of drought stress severity in isohydric hygrophylic plants. The best-fit analysis reported in **Figure 1** shows a highly significant exponential decay of Iso_i_ with increasing *g*_s_ (**Figure 1A**), because drought stress strongly enhances Iso_i_ for *g*_s_ < 200 mmol m^-2^ s^-1^, whereas Iso_i_ is unresponsive to higher *g*_s_. The severity of drought also significantly correlates with the investment of freshly assimilated carbon (C_iso_) to Iso_s_ (**Figure 1B**), indicating that a growing fraction of photosynthetic carbon sustains isoprene formation when the stress severely reduces *g*_s_. C_iso_ has also been widely shown to positively correlate with the unbalance between the ETR and *A*_N_ (Morfopoulos et al., 2014). In fact, ETR/*A*_N_ often increases as drought become more severe, especially when *A*_N_ is constrained by diffusional limitations (at stomatal or mesophyll level) rather than by biochemical limitations, as observed in fast-growing mesophytes, which are usually strong isoprene emitters (Loreto et al., 2014; Haworth et al., 2016). In contrast, there is a poor correlation between Iso_e_ and *g*_s_ (**Figure 1C**). In our survey, Iso_e_ is almost unresponsive to mild and moderate drought-induced depressions in *g*_s_, in both high (*Platanus × acerifolia, Populus nigra, P. deltoides*, on average Iso_e_ of 40.0 nmol m^-2^ s^-1^) and low isoprene emitters (*Eucalyptus occidentalis, Nicotiana tabacum*, on average Iso_e_ of 4.0 nmol m^-2^ s^-1^). In species with intermediate isoprene emission rates (on average Iso_e_ of 10.8 nmol m^-2^ s^-1^), instead, Iso_e_ either declines (*P. alba, E. citriodora*) or increases (*Moringa oleifera*) following drought-induced depression of *g*_s_. Data of our meta-analysis may help explain why isoprene emission fails representing the intensity of drought and heat stresses in current models (Harrison et al., 2013; Morfopoulos et al., 2013; Sharkey and Monson, 2014). We conclude that Iso_i_ and C_iso_, representing isoprene accumulation inside leaves, may allow better estimation than isoprene emission of the functional responses of plants to stress. However, we are aware that accuracy of *g****_s_*** measurements is inherently low for *g*_s_ < 20 mmol m^-2^ s^-1^ so that calculations of Iso_i_ (and to less extent of C_iso_ as well) have to be taken with some caution at very severe drought. The issue is of interest and merits further investigation.

## Author Contributions

AF wrote the first and second sections of the manuscript; CB wrote the second and third sections of the manuscript and conducted to the meta-analysis of data; FL contributed to manuscript writing and carefully reviewed the manuscript; MC and FF carefully reviewed the manuscript; MT drafted the manuscript and wrote section three and four of the manuscript.

## Conflict of Interest Statement

The authors declare that the research was conducted in the absence of any commercial or financial relationships that could be construed as a potential conflict of interest.
